# Euthyroid sick syndrome and its association with complications of type 1 diabetes mellitus onset

**DOI:** 10.1038/s41390-023-02494-5

**Published:** 2023-01-31

**Authors:** Pierluigi Marzuillo, Dario Iafusco, Stefano Guarino, Anna Di Sessa, Angela Zanfardino, Alessia Piscopo, Caterina Luongo, Daniela Capalbo, Martina Verde, Francesca Aiello, Adalgisa Festa, Emanuele Miraglia del Giudice, Anna Grandone

**Affiliations:** Department of Woman, Child and of General and Specialized Surgery, Università degli Studi della Campania “Luigi Vanvitelli”, Via Luigi De Crecchio 2, 80138 Napoli, Italy

## Abstract

**Objective:**

To evaluate (i) the prevalence and association of euthyroid sick syndrome (ESS) [decreased FT3 and/or FT4 and normal/decreased TSH] with severity indexes of type 1 diabetes mellitus (T1DM) onset such as diabetic ketoacidosis (DKA) and kidney damage [acute kidney injury (AKI) based on KDIGO criteria, acute tubular necrosis (ATN), renal tubular damage (RTD)], (ii) relationship between clinical/metabolic parameters at T1DM onset and thyroid hormones, and (iii) ESS as a prognostic indicator of delayed recovery from kidney damage.

**Methods:**

A total of 161 children with T1DM onset were included. RTD was defined by abnormal urinary beta-2-microglobulin and/or neutrophil gelatinase-associated lipocalin (NGAL) and/or tubular reabsorption of phosphate <85% and/or fractional excretion of Na>2%. ATN was defined by RTD+AKI.

**Results:**

Of 161 participants, 60 (37.3%) presented ESS. It was more prevalent in case of more severe T1DM presentation both in terms of metabolic derangement (DKA) and kidney function impairment (AKI, RTD and ATN). Only ATN, however, was associated with ESS at adjusted analysis. FT3 inversely correlated with serum triglycerides and creatinine, and urinary calcium/creatinine ratio and NGAL. Participants with euthyroidism showed earlier recovery from AKI than those with ESS. ESS spontaneously disappeared.

**Conclusions:**

ESS is associated with T1DM onset severity and spontaneously disappears. ESS delayed the recovery from AKI.

**Impact:**

- This is the first longitudinal study describing in detail the relationship between clinical/metabolic factors at type 1 diabetes mellitus (T1DM) onset and thyroid hormones, with particular attention to the relationship between diabetic ketoacidosis (DKA)-related kidney function impairment and euthyroid sick syndrome (ESS).
- Participants with more severe T1DM onset presentation both in terms of metabolic derangement and kidney function impairment had an increased prevalence of ESS.
- Children with ESS had a slower recovery from acute kidney injury compared with those without ESS.
- ESS spontaneously disappeared in all participants.

## Introduction

The euthyroid sick syndrome (ESS) also known as nonthyroidal illness syndrome is a condition characterized by a derangement in thyroid function tests, especially represented by low triiodothyronine (T3) and free T3 (FT3) concentrations.^1^ The ESS is a transient condition and it is more frequent in patients with severe critical illness, both in adulthood and childhood, and is associated with negative outcomes.^1,2^

Type 1 diabetes mellitus (T1DM) is a chronic disease characterized by insulin deficiency due to pancreatic beta-cell loss and leads to hyperglycemia.^3^ The onset of T1DM can manifest with a variable range of severity from mild clinical presentation to diabetic ketoacidosis (DKA) which can occur in 30–50% of children with newly diagnosed T1DM.^3,4^

In case of more severe onsets of T1DM –especially in case of more severe dehydration or DKA– acute kidney injury (AKI) can occur.^5,6^ AKI can manifest in up to 65.2% of children with DKA and in up to 21.1% of children without DKA at T1DM onset.^5,6^ Moreover, in 73.5% of the children with T1DM onset signs of renal tubular damage (RTD) can be detected.^5,6^

In the AKI pathophysiological mechanism, the first step is represented by an acute tubular damage (ATD). ATD is characterized by damage to the cells of the renal tubules which determines the loss of solutes in the urines. AKI occurs as a consequence of the ATD by vasoconstriction of the afferent arterioles which determines a fall in the glomerular filtration rate in order to reduce the solutes loss in the urine. This mechanism, however, could lead to persisting renal ischemia that shifts the AKI from functional to intrinsic, so determining acute tubular necrosis (ATN), expression of very severe kidney damage.^7^ This has relevance in children with T1DM because they already present an increased risk of diabetic nephropathy and the AKI could further increase this risk.^8,9^

In retrospective studies, it has been shown that children with DKA can manifest ESS in about 57% of cases^10,11^ and that the probability of developing ESS increases with the increase of acidosis. Moreover, an inverse relationship between acidosis and concentrations of free thyroid hormones has been found.^10,11^ Unlikely, no follow-up data of these patients are available.

We hypothesized that the prevalence of ESS increases with the increase of the severity of the T1DM presentation and that an association between ESS and impaired kidney function at T1DM onset could exist. Taking advantage of perspective data collection of the DiAKIdney (T1DM and AKI) cohort^6^ we aimed to evaluate (i) the prevalence and association of ESS with severity indexes of T1DM onset such as DKA and kidney damage (AKI, ATN, RTD), (ii) the relationship between clinical/metabolic parameters at T1DM onset and thyroid hormones, and (iii) the role of ESS as a prognostic indicator of delayed recovery from kidney damage.

## Methods

The DiAKIdney cohort was consecutively enrolled between December 2017 and August 2019.^6^ The study was approved by Research Ethical Committee of Università degli Studi della Campania “Luigi Vanvitelli” (approval number 368) and all parents provided written informed consent. As previously described, participants with the onset of T1DM with age <18 years and not on any medication apart intravenous 0.9% NaCl infusion were enrolled. Participants assuming any medication for any chronic condition before the T1DM onset or not returning for the scheduled follow-up or with congenital anomalies of the kidney and urinary tract were excluded.^6^

All the participants had T1DM autoimmune diabetes with glutamic acid decarboxylase and/or islet antigen two and/or insulin and/or Zinc transporter eight antibodies positive at the time of diagnosis. All the participants needed to be on insulin consistently following diagnosis.

After discharge, all the participants were followed up after 14 days. The participants not having shown recovery from AKI or RTD at this time returned again after 30 days and—in case of persistence of damage—after 60 days.^6^

Data regarding age, weight, height, body mass index (BMI) with its standard deviation score (SDS), blood pressure (BP), heart rate (HR), urinary output for the first 24 h, renal ultrasound, full blood count, baseline blood glucose concentration, serum creatinine (measured by the Jaffe method),^6^ ketones, Na, Cl, phosphorus, blood pH, bicarbonates, glycate hemoglobin (Hb1Ac) and a urine sample for urine dipstick, urinary proteins, microalbuminuria, creatinine, sodium, calcium, phosphate, beta-2-microglobulin as well as neutrophil gelatinase-associated lipocalin (NGAL) both at T1DM onset and at follow-up visits were collected.^6^

In addition to these data, in this manuscript, we used also prospectively collected data about thyroid-stimulating hormone (TSH), free thyroxine (FT4) and free triiodothyronine (FT3) concentrations at T1DM onset. In all the participants also antithyroglobulin and anti-thyroid peroxidase antibodies were dosed. The thyroid hormones were dosed again after 6–12 months.

The original DiAKIdney cohort comprehended 185 participants. Among these participants, 1 was excluded because presenting with autoimmune thyroiditis and overt severe hypothyroidism and 23 because data about thyroid hormones at T1DM onset were not available.

### Definitions

Euthyroidism was defined when FT3, FT4 and TSH were respectively within the following ranges: FT3 2.3–5.5 pg/mL, FT4 9.3–17.1 pg/mL, TSH 0.27–4.2 mcIU/mL.^11^ ESS was defined when FT3 and/or FT4 were decreased and TSH concentrations were normal or decreased.^11^

DKA was defined by blood glucose level ≥200 mg/dL, pH ≤7.3 or bicarbonates ≤15 mEq/L, and elevation of serum ketones.^5^ According to ISPAD clinical practice consensus guidelines, DKA was classified as mild if venous pH <7.3 or serum bicarbonate <15 mmol/L, moderate if pH <7.2 and serum bicarbonate <10 mmol/L, severe if pH <7.1 and serum bicarbonate <5 mmol/L.^12^

AKI was defined according to the Kidney Disease/Improving Global Outcomes (KDIGO) serum creatinine and/or urine output criteria.^13^ In brief, stage 1 AKI is defined by serum creatinine 1.5–1.9 times baseline or urine output <0.5 mL/kg/h for 6–12 h, stage 2 AKI by serum creatinine 2.0–2.9 times baseline or urine output <0.5 mL/kg/h for ≥12 h, stage 3 AKI by serum creatinine 3.0 times baseline or urine output <0.3 mL/kg/h for ≥24 h or anuria for ≥12 h. None of the participants presented stage 3 AKI nor required hemodialysis; therefore, we considered the participants with stage 2 AKI as affected by severe AKI for the analyses of this manuscript.

We considered as basal the creatinine values obtained at the last follow-up when all the biochemical parameters showed normalization and estimated glomerular filtration rate (eGFR) was within the normal range by age.^14^ Prerenal-AKI (P-AKI) was defined by KDIGO creatinine and/or urine output criteria associated with FeNa <1%.^15^

In general, we considered the participants as having RTD if presenting with one or more abnormal values among urinary levels of beta-2-microglobulin >0.33 mg/L, neutrophil gelatinase-associated lipocalin (NGAL) values >95th percentile by age,^16^ TRP <85%, and FeNa >2%.

TRP was calculated as follows: TRP(%) = 1 – [(Urinary phosphorus/Serum phosphorus) × (Serum creatinine/Urinary creatinine].^17^

ATD was defined by urinary levels of beta-2-microglobulin >0.33 mg/L^18^ and/or NGAL values >95th percentile by age^16^ and/or TRP <85%^17,19^ and/or FeNa >2% in the absence of AKI according to KDIGO criteria.^13^

ATN was defined by (i) the presence of AKI according to KDIGO criteria^13^ and FENa >2%^15^ or (ii) the presence of AKI, FENa between 1 and 2%, and/or urinary levels of beta-2-microglobulin >0.33 mg/L^18^ and/or neutrophil gelatinase-associated lipocalin (NGAL) values >95th percentile by age^16^ and/or TRP <85%.^17,19^

FeNa was calculated as follows: FeNa(%) = (Urinary sodium/Urinary creatinine) / (Serum sodium/Serum creatinine).^15^

Recovery from RTD was defined by normalization of the investigated physiological and biochemical parameters. AKI resolution was defined by a complete reversal of AKI.^20^

Levels of cholesterol and triglycerides were considered within the normal range if <200 and <125 mg/dL, respectively.^21,22^

### Statistical analysis

*P* values <0.05 were considered significant. Differences for continuous variables were analyzed with the independent-sample *t*-test for normally distributed variables and with the Mann–Whitney test in case of non-normality. Qualitative variables were compared using the *χ*^2^ test.

Pearson and Spearman correlation tests were applied to evaluate associations of all linear data with FT3, FT4 or TSH. These variables were grouped into clinical, serum and urinary factors in order to give useful information for each of the steps of usual evaluations of patients.

For univariate analysis, the Pearson test was used for parametric while the Spearman test for nonparametric variables.

After Bonferroni correction, the variables resulting significant at univariate analyses were included in multiple linear regression analyses to identify the clinical, serum and urinary factors independently correlating with FT3, FT4 or TSH.

Finally, the clinical, serum and urinary variables persisting significant after Bonferroni correction at each (clinical, serum, and urinary) multiple regression analysis were included in a unique final model. In the multiple linear regression analyses the nonparametric variables were log-transformed.

The variables included in the univariate analyses, in the multivariate analyses and then in the final model are specified in Tables 2–4 and in the footnotes of these tables.

Logistic regression models were used with the aim of exploring associations with ESS of the dichotomic variables. These variables were a priori chosen on the basis of the factors previously associated with the severity of T1DM onset.^6^ We added in the multivariate logistic regression analyses the parameters that associated with ESS at univariate logistic regression analysis after Bonferroni correction was applied.

The variables included firstly in the univariate and then in the multivariate logistic regression analysis are shown in Table 5 and in the footnotes of this table.

The time to AKI and RTD recovery in participants with euthyroidism and ESS was studied by survival analysis according to the Kaplan–Meier method. The day of admission was considered as the starting point, while the end point was the date of AKI or RTD resolution. Kaplan–Meier curves were compared by log-rank test.

The Stat-Graph XVII software for Windows was used for all statistical analyses with the exception of logistic regression models made with SPSS 25 software for Windows and Kaplan–Meier analysis made with GraphPad Prism 8 for Windows.

## Results

### General characteristics

One hundred and sixty-one children with T1DM onset were enrolled. The mean age was 9.1 years (4.0 SDS). Out of 161 participants, DKA, AKI, and RTD were found in 85 (52.7%), 73 (45.3%), and 120 (74.5%) participants, respectively.

More in detail, regarding the kidney involvement of the enrolled population, we found ATN in 55 (34.2%), P-AKI in 18 (11.2%), ATD in 48 (29.8%) and absence of kidney involvement in 40 (24.8%) participants.

ESS was found in 60 out of 161 participants (37.3%). More in detail in 49 out of 85 participants (57.6%) with DKA and in 11 out of 76 (14.5%) without DKA at T1DM onset (*p* < 0.001). ESS was more prevalent among participants with AKI, RTD and ATN compared respectively with participants without AKI, RTD and ATN (Supplementary Fig. S1). As expected, among participants with the absence of kidney involvement, the prevalence of ESS was lower compared with others (Supplementary Fig. S1).

ESS disappeared in all participants when thyroid hormones were repeated after a median of 12 months from T1DM onset (range: 6–12 months).

Participants with ESS at T1DM onset showed older age; lower BMI-SDS, lymphocytes, phosphorus, bicarbonates, eGFR, and TRP levels; higher weight loss, HR, HbA1c, hemoglobin, cholesterol, triglycerides, chloride, ketones, creatinine, highest creatinine/basal creatinine (HC/BC) ratio, UPr/Cr, microalbuminuria, NGAL, beta-2-microglobulin, FeNa, and UCa/Cr levels; lower prevalence of family history of T1DM; and higher prevalence of HR >2 SDS, DKA, AKI, ATN, RTD and proteinuria compared with participants with euthyroidism (Table 1).

**Table 1:** Clinical and laboratory characteristics of all enrolled patients and of the patients with and without ESS at T1DM onset.

		At T1DM onset			Data obtained after recovery from the acute phase and normalization of the kidney/tubular damage (in patients having presented this damage)		
	All patients, *N* = 161	Euthyroidism, *N* = 101	ESS, *N* = 60	*p*	Euthyroidism, *N* = 101	ESS, *N* = 60	*p*
Age, years, mean (SDS)	9.1 (4.0)	8.6 (4.1)	9.9 (3.9)	0.047	–	–	–
Male sex, *N* (%)	74 (46.0)	47 (46.5)	27 (45.0)	0.85	–	–	–
Weight loss, %, median (IQR)	7.3 (8.4)	5.3 (8.1)	8.6 (7.7)	<0.001	–	–	–
Polyuria and polydipsia duration, days, median (IQR)	14.0 (8.0)	10.0 (8.0)	14.0 (13.0)	0.18	–	–	–
Family history of T1DM, *N* (%)	21 (13.0)	20 (19.8)	1 (1.7)	0.001	–	–	–
BMI, SDS, median (IQR)	–0.37 (2.0)	–0.17 (2.0)	–0.80 (2.2)	0.003	0.36 (1.57)	0.01 (1.1)	<0.001
SBP, SDS, mean (SDS)	0.6 (0.9)	0.61 (0.81)	0.44 (0.94)	0.22	–0.3 (0.9)	–0.4 (0.8)	0.38
DBP, SDS, mean (SDS)	0.4 (0.8)	0.27 (0.85)	0.51 (0.84)	0.09	–0.19 (0.72)	–0.13 (0.64)	0.56
HR, beats/min, mean (SDS)	111.0 (21.3)	108.5 (21.8)	121.2 (18.0)	<0.001	87.7 (15.6)	86.6 (13.9)	0.63
HR >2 SDS for age and gender, *N* (%)	59 (36.6)	26 (25.7)	33 (55.0)	<0.001	0	0	0.99
Kussmaul, *N* (%)	28 (17.4)	16 (15.8)	12 (20)	0.50	–	–	–
Coma, *N* (%)	22 (13.7)	13 (12.9)	9 (15)	0.70	–	–	–
DKA, *N* (%)^a^	85 (52.7)	36 (35.6)	49 (81.7)	<0.001	–	–	–
Hemoglobin, g/dL, mean (SDS)	14.1 (1.4)	13.8 (1.3)	14.5 (1.6)	0.003	12.8 (0.8)	12.9 (1.0)	0.76
WBC, n/mcL, median (IQR)	8550 (4110)	8470 (3490)	11783 (5045)	0.34	7092 (3220)	6911 (1920)	0.35
Neutrophils, n/mcL, median (IQR)	4700 (3380)	4350 (2900)	5050 (5000)	0.08	2890 (1720)	3050 (1610)	0.70
Lymphocytes, n/mcL, median (IQR)	2900 (1800)	3100 (1400)	2450 (2050)	0.001	2650 (1320)	2410 (820)	0.17
Platelets, n/mcL, median (IQR)	296,000 (136,000)	300,000 (131,000)	295,500 (117,000)	0.84	295,000 (106,000)	313,500 (119,000)	0.78
Cholesterol, mg/dL, median (IQR)	159.0 (52.0)	153.0 (31.0)	189.5 (94.5)	<0.001	159.0 (44)	187.0 (57.5)	<0.001
Abnormal cholesterol levels, *N* (%)	36 (22.4)	13 (12.9)	26 (43.3)	<0.001	10 (9.9)	19 (31.7)	<0.001
Triglycerides, mg/dL, median (IQR)	126.5 (133.5)	100.0 (63.5)	211.5 (301.5)	<0.001	103 (75.0)	110.5 (59.0)	0.27
Abnormal triglycerides levels, *N* (%)	78 (48.4)	32 (32)	46 (76.7)	<0.001	31 (30.7)	18 (30)	0.92
Serum phosphorus levels, mg/dL, mean (SDS)	3.7 (0.9)	3.9 (1.0)	3.4 (0.8)	<0.001	4.6 (0.7)	4.7 (0.5)	0.16
Serum bicarbonate levels, mEq/L, mean (SDS)^b^	16.2 (6.7)	18.3 (6.7)	12.7 (6.8)	<0.001	–	–	–
Serum ketones, mmol/L, mean (SDS)	4.3 (2.8)	3.5 (2.8)	5.7 (2.2)	<0.001	–	–	–
Corrected serum Na level, mEq/L, median (IQR)	140.0 (3.8)	140.0 (3.5)	140.6 (3.9)	0.29	138 (3.0)	139 (2.0)	0.4
Serum chloride levels, mEq/L, median (IQR)	103.0 (5.0)	102.0 (5.0)	104.0 (6.5)	0.02	104 (2.0)	105 (3.0)	0.28
HbA1c, %, mean (SDS)	11.5 (2.0)	11.0 (2.0)	12.2 (1.8)	0.005	–	–	–
eGFR, mL/min/1.73 m^2^, mean (SDS)	94.8 (26.3)	98.9 (24.4)	87.8 (28.1)	0.009	127.4 (16.9)	130.1 (18.8)	0.23
Creatinine, mg/dL, median (IQR)	0.79 (0.29)	0.74 (0.17)	0.89 (0.47)	<0.001	0.57 (0.1)	0.59 (0.07)	0.13
Diuresis (first 6 h), mL/kg/h, median (IQR)	1.2 (0.87)	1.1 (0.72)	1.2 (0.85)	0.29	–	–	–
AKI, *N* (%)	73 (45.3)	33 (32.7)	40 (66.7)	<0.001	–	–	–
HC/BC ratio, median (IQR)	1.4 (0.48)	1.3 (0.35)	1.6 (0.64)	<0.001	–	–	–
RTD, *N* (%)	120 (74.5)	64 (63.4)	56 (93.3)	<0.001	–	–	–
P-AKI, *N* (%)	18 (11.2)	14 (13.9)	4 (10)	0.20	–	–	–
ATD, *N* (%)	48 (29.8)	32 (31.7)	16 (26.7)	0.50	–	–	–
ATN, *N* (%)	55 (34.2)	19 (18.8)	36 (60)	<0.001	–	–	–
UPr/Cr, mg/mg, median (IQR)	0.41 (0.48)	0.3 (0.36)	1.0 (1.0)	<0.001	0.15 (0.08)	0.17 (0.10)	0.40
Microalbuminuria, mg/L, median (IQR)	24.5 (56.5)	19.5 (39.5)	47.0 (92.5)	0.002	5.0 (2.0)	5.0 (3.0)	0.9
Proteinuria, *N* (%)	128 (79.5)	69 (68.3)	59 (98.3)	<0.001	20 (19.8)	17 (28.3)	0.21
Urinary NGAL, ng/mL, median (IQR)	88.1 (210.7)	24.3 (117.8)	220.6 (188.6)	<0.001	4.6 (6.5)	10.2 (17.2)	<0.001
Urinary Beta-2-microglobulin, mg/L, median (IQR)	1.1 (3.9)	0.2 (3.9)	4.0 (2.2)	<0.001	0.06 (0.08)	0.06 (0.09)	0.53
FeNa, %, median (IQR)	1.1 (1.4)	0.69 (1.1)	1.6 (1.2)	<0.001	0.79 (0.78)	0.90 (0.88)	0.40
TRP, %, median (IQR)	78.0 (45.0)	84.0 (18.0)	50.5 (59.5)	<0.001	91.0 (7.0)	90.2 (5.0)	0.35
UCa/Cr, mg/mg, median (IQR)	0.63 (0.98)	0.37 (0.55)	1.2 (0.93)	<0.001	0.13 (0.13)	0.11 (0.10)	0.15
Abnormal renal echogenicity, *N* (%)	41 (25.5)	21 (20.8)	20 (33.3)	0.07	0	0	0.99
Renal length, SDS, median (IQR)	1.1 (1.8)	0.94 (1.8)	1.3 (1.8)	0.29	0.49 (1.6)	0.92 (1.7)	0.36

*AKI* acute kidney injury, *BMI* body mass index, *DBP* diastolic blood pressure, *DKA* diabetic ketoacidosis, *eGFR* estimated glomerular filtration rate, *HbA1c* glycate hemoglobin, *HC/BC* highest creatinine/basal creatinine, *HR* hearth rate, *NGAL* neutrophil gelatinase-associated lipocalin, *SBP* systolic blood pressure, *SDS* standard deviation score, *T1DM* type 1 diabetes mellitus, *UPr/Cr* urinary protein:creatinine ratio.

^a^Among patients with DKA, 56 out of 85 patients (65.8%) presented AKI.

^b^The mean serum bicarbonate level was 10.9 mmol/L (4.2 SDS) in the population of 85 patients with DKA.

At follow-up evaluation, participants with ESS at T1DM onset presented lower BMI-SDS, higher cholesterol levels, higher prevalence of hypercholesterolemia, and higher NGAL levels (NGAL always within the range of normality) compared with participants with euthyroidism (Table 1).

### Factors correlating and independently influencing FT3 levels

Among clinical factors, FT3 negatively correlated with weight loss and HR (Table 2). Among serum factors, FT3 positively correlated with serum phosphorus, serum bicarbonates and eGFR levels while negatively correlated with hemoglobin, cholesterol, triglycerides, ketones, HbA1c, HC/BC ratio, and creatinine levels (Table 2). Among urinary factors, FT3 positively correlated with TRP while negatively correlated with UPr/Cr, microalbuminuria, NGAL, beta-2-microglobulin, FeNa, UCa/Cr (Table 2).

**Table 2:** Univariate and multivariate analysis of factors correlating with FT3.

		Univariate		Multivariate^a^			Final multivariate^a^		
		*r*	*p*^d^	*r*	*F*-ratio	*p*^f^	*r*	*F*-ratio	*p*^d^
Clinical factors	Weight loss^c^, %	–0.34	**<0.001**	–0.42	6.6	**0.01**	0.08	0.3	0.6
	Polyuria and polydipsia duration^c^, days	–0.18	0.02	–	–	–	–	–	–
	BMI^c^, SDS	0.19	0.02	–	–	–	–	–	–
	SBP^b^, SDS	0.1	0.22	–	–	–	–	–	–
	DBP^b^, SDS	–0.11	0.15	–	–	–	–	–	–
	HR^b^, beats/min	–0.38	**<0.001**	–0.01	13.6	**<0.001**	–0.01	1.4	0.2
	Diuresis (first 6 h)^c^, mL/kg/h	0.007	0.93	–	–	–	–	–	–
				*R*-square = 0.16 *R*-square (adjusted) = 0.15 Model *p* < 0.001			See below		

		Univariate		Multivariate^a^			Final multivariate^a^		
		*r*	*p*^e^	*r*	*F*-ratio	*p*^d^	*r*	*F*-ratio	*p*^d^
Serum factors	Hemoglobin^b^, g/dL	–0.27	**<0.001**	0.008	0.03	0.87	–	–	–
	WBC^c^, n/mcL	–0.12	0.13	–	–	–	–	–	–
	Neutrophils^c^, n/mcL	–0.21	0.009	–	–	–	–	–	–
	Lymphocytes^c^, n/mcL	0.16	0.04	–	–	–	–	–	–
	Platelets^c^, n/mcL	–0.005	0.95	–	–	–	–	–	–
	Cholesterol^c^, mg/dL	–0.35	**<0.001**	–0.21	0.11	0.74	–	–	–
	Triglycerides^c^, mg/dL	–0.53	**<0.001**	–0.82	10.2	**0.002**	–0.7	12.2	**0.001**
	Serum phosphorus levels^b^, mg/dL	0.36	**<0.001**	0.07	0.82	0.36	–	–	–
	Serum bicarbonate level^b^, mEq/L	0.49	**<0.001**	0.03	6.93	**0.007**	–0.03	3.8	0.05
	Serum ketones^b^, mmol/L	–0.43	**<0.001**	n.a.^g^	n.a.^g^	n.a.^g^	–	–	–
	Corrected serum Na level^c^, mEq/L	–0.03	0.74	–	–	–	–	–	–
	Serum chloride levels^c^, mEq/L	–0.17	0.03	–	–	–	–	–	–
	HbA1c^b^, %	–0.36	**<0.001**	–0.03	0.78	0.38	–	–	–
	eGFR^b^, mL/min/1.73 m^2^	0.29	**<0.001**	n.a.^h^	n.a.^h^	n.a.^h^	–	–	–
	Creatinine^c^, mg/dL	–0.44	**<0.001**	–0.84	10.0	**0.002**	–1.6	11.5	**0.001**
	HC/BC ratio^c^	–0.43	**<0.001**	n.a.^h^	n.a.^h^	n.a.^h^	–	–	–
				*R*-square = 0.43 *R*-square (adjusted) = 0.40 Model *p* < 0.001			See below		

		Univariate		Multivariate^a^			Final multivariate^a^		
		*r*	*p*^d^	*r*	*F*-ratio	*p*^d^	*r*	*F*-ratio	*p*^d^
Urinary factors	UPr/Cr^c^, mg/mg	–0.51	**<0.001**	–0.13	0.31	0.58	–	–	–
	Microalbuminuria^c^, mg/L	–0.34	**<0.001**	–0.05	0.13	0.72	–	–	–
	Urinary NGAL^c^, ng/mL	–0.58	**<0.001**	–0.34	7.2	**0.007**	–0.36	10.9	**0.001**
	Beta-2-microglobulin^c^, mg/L	–0.49	**<0.001**	–0.009	0.02	0.88	–	–	–
	FeNa^c^, %	–0.27	**0.001**	0.40	0.07	0.79	–	–	–
	TRP^c^, %	0.47	**<0.001**	0.06	0.08	0.78	–	–	–
	UCa/Cr^c^, mg/mg	–0.57	**<0.001**	–0.62	9.4	**0.003**	–0.64	13.6	**<0.001**
				*R*-square = 0.40 *R*-square (adjusted) = 0.37 Model *p* < 0.001			*R*-square = 0.51 *R*-square (adjusted) = 0.48 Model *p* < 0.001		

^a^After Bonferroni correction, the variables resulting significant at univariate analyses were included in multivariate analyses to identify the clinical, serum and urinary factors independently correlating with FT3. Finally, the clinical, serum and urinary variables persisting significant after Bonferroni correction at each (clinical, serum, and urinary) multivariate analysis were included in a unique final multivariate analysis. The variables included in these models are indicated by the presence of the results of the analysis in the columns “*r*”, “*F*-ratio” and “*p*”. The excluded variables are indicated by “–” in the columns “*r*”, “*F*-ratio” and “*p*”.

Pearson^b^ and Spearman^c^ correlation tests were applied to evaluate associations of parametric and nonparametric data, respectively.

^c^Log-transformed at multivariate analysis.

^d^After Bonferroni correction was considered significant a *p* ≤ 0.007.

^e^After Bonferroni correction was considered significant a *p* ≤ 0.003.

^f^After Bonferroni correction was considered significant a *p* ≤ 0.025.

^g^Ketones not evaluated in the multivariate analysis because clinically related to bicarbonates and with a less strong association with FT3 compared with bicarbonates (on the basis of the *r* value at univariate analysis).

^h^eGFR and HC/BC ratio not evaluated in the multivariate analysis because clinically related to creatinine and with a less strong association with FT3 compared with creatinine (on the basis of the *r* value at univariate analysis).

At multivariate analysis, the association of FT3 with weight loss and HR among clinical factors, with triglycerides, bicarbonates and creatinine levels among serum factors, and NGAL and UCa/Cr among urinary factors persisted significant (Table 2).

Adding all the clinical, serum and urinary factors significantly correlated with FT3 at multivariate analysis in a final multivariate model, a significant association of FT3 with serum triglycerides, serum creatinine, urinary NGAL and UCa/Cr was found (Table 2).

The relation between these independent parameters and FT3 is shown in Supplementary Fig. S2.

### Factors correlating and independently influencing FT4 levels

Among clinical factors, FT4 negatively correlated with weight loss and HR (Table 3). Among serum factors, FT4 positively correlated with serum bicarbonates and eGFR levels while negatively correlated with cholesterol, triglycerides, ketones, HC/BC ratio, and creatinine levels (Table 3).

**Table 3:** Univariate and multivariate analysis of factors correlating with FT4.

		Univariate		Multivariate^a^				Final multivariate^a^		
		*r*	*p*^d^	*r*		*F*-ratio	*p*^f^	*r*	*F*-ratio	*p*^f^
Clinical factors	Weight loss^c^, %	–0.29	**<0.001**	–1.2		6.4	**0.01**	–0.38	0.58	0.45
	Polyuria and polydipsia duration^c^, days	–0.07	0.35	–		–	–	–	–	–
	BMI^c^, SDS	0.17	0.03	–		–	–	–	–	–
	SBP^b^, SDS	0.15	0.06	–		–	–	–	–	–
	DBP^b^, SDS	–0.15	0.06	–		–	–	–	–	–
	HR^b^, beats/min	–0.23	**0.007**	–0.02		2.9	0.09	–	–	–
	Diuresis (first 6 h)^b^, mL/kg/h	–0.09	0.27	–		–	–	–	–	–
				*R*-square = 0.08 *R*-square (adjusted) = 0.07 Model *p* = 0.002				See below		

		Univariate		Multivariate^a^				Final multivariate^a^		
		*r*	*p*^e^	*r*		*F*-ratio	*p*^i^	*r*	*F*-ratio	*p*^*f*^
Serum factors	Hemoglobin^b^, g/dL	–0.07	0.38	–		–	–	–	–	–
	WBC^c^, n/mcL	–0.11	0.17	–		–	–	–	–	–
	Neutrophils^c^, n/mcL	–0.10	0.16	–		–	–	–	–	–
	Lymphocytes^c^, n/mcL	–0.08	0.29	–		–	–	–	–	–
	Platelets^c^, n/mcL	–0.02	0.76	–		–	–	–	–	–
	Cholesterol^c^, mg/dL	–0.26	**0.001**	–3.9		4.8	0.03	–	–	–
	Triglycerides^c^, mg/dL	–0.35	**<0.001**	–0.94		1.5	0.22	–	–	–
	Serum phosphorus levels^b^, mg/dL	0.19	0.01	–		–	–	–	–	–
	Serum bicarbonate level^b^, mEq/L	0.41	**<0.001**	0.09		7.6	**0.007**	0.13	18.3	**<0.001**
	Serum ketones^b^, mmol/L	–0.28	**<0.001**	n.a.^g^		n.a.^g^	n.a.^g^	–	–	–
	Corrected serum Na level^c^, mEq/L	–0.03	0.68	–		–	–	–	–	–
	Serum chloride levels^c^, mEq/L	–0.19	0.02	–		–	–	–	–	–
	HbA1c^b^, %	–0.18	0.02	–		–	–	–	–	–
	eGFR^b^, mL/min/1.73 m^2^	0.33	**<0.001**	n.a.^h^		n.a.^h^	n.a.^h^	–	–	–
	Creatinine^c^, mg/dL	–0.28	**<0.001**	n.a.^h^		n.a.^h^	n.a.^h^	–	–	–
	HC/BC ratio^c^	–0.34	**<0.001**	–2.4		1.9	0.17	–	–	–
				*R*-square = 0.26 *R*-square (adjusted) = 0.24 Model *p* < 0.001				See below		

		Univariate		Multivariate^a^				Final multivariate^a^		
		*r*	*p*^d^	*r*		*F*-ratio	*p*^j^	*r*	*F*-ratio	*p*^f^
Urinary factors	UPr/Cr^c^, mg/mg	–0.36	**<0.001**	–1.1		2.5	0.12	–	–	–
	Microalbuminuria^c^, mg/L	–0.18	0.02	–		–	–	–	–	–
	Urinary NGAL^c^, ng/mL	–0.35	**<0.001**	–0.48		1.6	0.21	–	–	–
	Beta-2-microglobulin^c^, mg/L	–0.32	**<0.001**	–0.08		0.16	0.69	–	–	–
	FeNa^c^, %	–0.23	**0.004**	–0.43		0.97	0.33	–	–	–
	TRP^c^, %	0.25	**0.001**	0.46		0.48	0.49	–	–	–
	UCa/Cr^c^, mg/mg	–0.32	**<0.001**	–0.01		0.001	0.98	–	–	–
				*R*-square = 0.2 *R*-square (adjusted) = 0.17 Model *p* < 0.001				*R*-square = 0.17 *R*-square (adjusted) = 0.16 Model *p* < 0.001		

^a^After Bonferroni correction, the variables resulting significant at univariate analyses were included in multivariate analyses to identify the clinical, serum and urinary factors independently correlating with FT4. Finally, the clinical, serum and urinary variables persisting significant after Bonferroni correction at each (clinical, serum, and urinary) multivariate analysis were included in a unique final multivariate analysis. The variables included in these models are indicated by the presence of the results of the analysis in the columns “*r*”, “*F*-ratio” and “*p*”. The excluded variables are indicated by “–” in the columns “*r*”, “*F*-ratio” and “*p*”.

Pearson^b^ and Spearman^c^ correlation tests were applied to evaluate associations of parametric and nonparametric data, respectively.

^c^Log-transformed at multivariate analysis.

^d^After Bonferroni correction was considered significant a *p* ≤ 0.007.

^e^After Bonferroni correction was considered significant a *p* ≤ 0.003.

^f^ After Bonferroni correction was considered significant a *p* ≤ 0.025.

^g^Ketones not evaluated in the multivariate analysis because clinically related to bicarbonates and with a less strong association with FT4 compared with bicarbonates (on the basis of the *r* value at univariate analysis).

^h^eGFR and creatinine not evaluated in the multivariate analysis because clinically related to HC/BC ratio and with a less strong association with FT4 compared with HC/BC ratio (on the basis of the *r* value at univariate analysis).

^i^After Bonferroni correction was considered significant a *p* ≤ 0.012.

^j^After Bonferroni correction was considered significant a *p* ≤ 0.008.

Among urinary factors, FT4 positively correlated with TRP while negatively correlated with UPr/Cr, NGAL, beta-2-microglobulin, FeNa, UCa/Cr (Table 3).

At multivariate analysis, the association of FT4 only with weight loss among clinical factors and bicarbonates levels among serum factors persisted significant (Table 3).

Adding these two parameters in a final multivariate model, a significant association of only FT4 with serum bicarbonates levels was found (Table 3).

The relation between this independent parameter and FT4 is shown in Supplementary Fig. S3.

### Factors correlating and independently influencing TSH levels

TSH did not correlate with any of the clinical and urinary factors (Table 4). Among serum factors, TSH negatively correlated with hemoglobin and WBC (Table 4).

**Table 4:** Univariate and multivariate analysis of factors correlating with TSH.

		Univariate		Multivariate^a^		
		*r*	*p*^d^	*r*	*F*-ratio	*p*^f^
Clinical factors	Weight loss^c^, %	–0.34	0.67	–	–	**–**
	Polyuria and polydipsia duration^c^, days	–0.17	0.03	–	–	–
	BMI^c^, SDS	–0.09	0.26	–	–	–
	SBP^b^, SDS	0.03	0.74	–	–	–
	DBP^b^, SDS	0.001	0.96	–	–	–
	HR^b^, beats/min	–0.05	0.54	–	–	**–**
	Diuresis (first 6 h)^c^, mL/kg/h	–0.09	0.24	–	–	–
				*R*-square = n.a. *R*-square (adjusted) = n.a Model *p* = n.a.		

		Univariate		Multivariate^a^		
		*r*	*p*^e^	*r*	*F*-ratio	*p*^f^
Serum factors	Hemoglobin^b^, g/dL	–0.24	**0.002**	–1.3	8.8	**0.003**
	WBC^c^, n/mcL	–0.26	**0.001**	–1.1	0.29	0.59
	Neutrophils^c^, n/mcL	–0.17	0.03	–	–	–
	Lymphocytes^c^, n/mcL	–0.21	0.007	–	–	–
	Platelets^c^, n/mcL	–0.16	0.04	–	–	–
	Cholesterol^c^, mg/dL	–0.10	0.19	–	–	–
	Triglycerides^c^, mg/dL	–0.11	0.15	–	–	–
	Serum phosphorus levels^b^, mg/dL	0.05	0.50	–	–	–
	Serum bicarbonate level^b^, mEq/L	–0.09	0.28	–	–	–
	Serum ketones^b^, mmol/L	0.04	0.62	–	–	–
	Corrected serum Na level^c^, mEq/L	0.04	0.64	–	–	–
	Serum chloride levels^c^, mEq/L	–0.10	0.22	–	–	–
	HbA1c^b^, %	–0.03	0.72	–	–	–
	eGFR^b^, mL/min/1.73 m^2^	–0.07	0.36	–	–	–
	Creatinine^c^, mg/dL	0.07	0.35	–	–	–
	HC/BC ratio^c^	0.008	0.91	–	–	–
				*R*-square = 0.06 *R*-square (adjusted) = 0.05 Model *p* = 0.008		

		Univariate		Multivariate^a^		
		*r*	*p*^d^	*r*	*F*-ratio	*p*
Urinary factors	UPr/Cr^c^, mg/mg	–0.13	0.10	–	–	–
	Microalbuminuria^c^, mg/L	–0.03	0.72	–	–	–
	Urinary NGAL^c^, ng/mL	–0.02	0.76	–	–	**–**
	Beta-2-microglobulin^c^, mg/L	–0.04	0.62	–	–	–
	FeNa^c^, %	0.08	0.29	–	–	–
	TRP^c^, %	0.009	0.91	–	–	–
	UCa/Cr^c^, mg/mg	–0.10	0.20	–	–	**–**
				*R*-square = n.a. *R*-square (adjusted) = n.a. Model *p* = n.a.		

^a^After Bonferroni correction, the variables resulting significant at univariate analyses were included in multivariate analyses to identify the clinical, serum and urinary factors independently correlating with TSH. Finally, the clinical, serum and urinary variables persisting significant after Bonferroni correction at each (clinical, serum, and urinary) multivariate analysis were included in a unique final multivariate analysis. In this case, a final multivariate analysis was not run because only one variable persisted significant in multivariate analysis. The variables included in these models are indicated by the presence of the results of the analysis in the columns “*r*”, “*F*-ratio” and “*p*”. The excluded variables are indicated by “–” in the columns “*r*”, “*F*-ratio” and “*p*”.

Pearson^b^ and Spearman^c^ correlation tests were applied to evaluate associations of parametric and nonparametric data, respectively.

^c^Log-transformed at multivariate analysis.

^d^After Bonferroni correction was considered significant a *p* ≤ 0.007.

^e^After Bonferroni correction was considered significant a *p* ≤ 0.003.

^f^After Bonferroni correction was considered significant a *p* ≤ 0.025.

In multivariate analysis, only a correlation with hemoglobin levels persisted (Table 4).

### Analysis of factors associated with ESS

In univariate logistic regression analysis, DKA, AKI, RTD and ATN were significantly associated with ESS (Table 5). This significance was maintained only by ATN at multivariate analysis (Table 5).

**Table 5:** Exploratory analysis of factors potentially associated with ESS.

	Univariate analysis			Multivariate analysis^a^		
Risk factors	OR	95% CI	*p*^b^	OR	95% CI	*p*^b^
>5% dehydration	2.8	1.4–5.8	**0.004**	1.8	0.8–4.3	0.16
Severe DKA	1.1	1.08–1.2	**<0.001**	2.5	1.1–5.6	0.04
Severe AKI	3.0	1.5–6.9	**0.009**	0.9	0.4–2.5	0.89
RTD	8.1	2.7–24.1	**<0.001**	0.5	0.1–1.6	0.21
ATN	6.5	3.1–13.3	**<0.001**	4.2	1.8–9.6	0.001

*AKI* acute kidney injury, *ATN* acute tubular necrosis, *CI* confidence interval, *DKA* diabetic ketoacidosis, *OR* odds ratio, *RTD* renal tubular damage.

^a^After Bonferroni correction, the variables resulting significant in univariate analyses were included in multivariate analysis.

^b^After Bonferroni correction was considered significant a *p* ≤ 0.01.

### Kaplan–Meier analysis

**Fig. 1:**
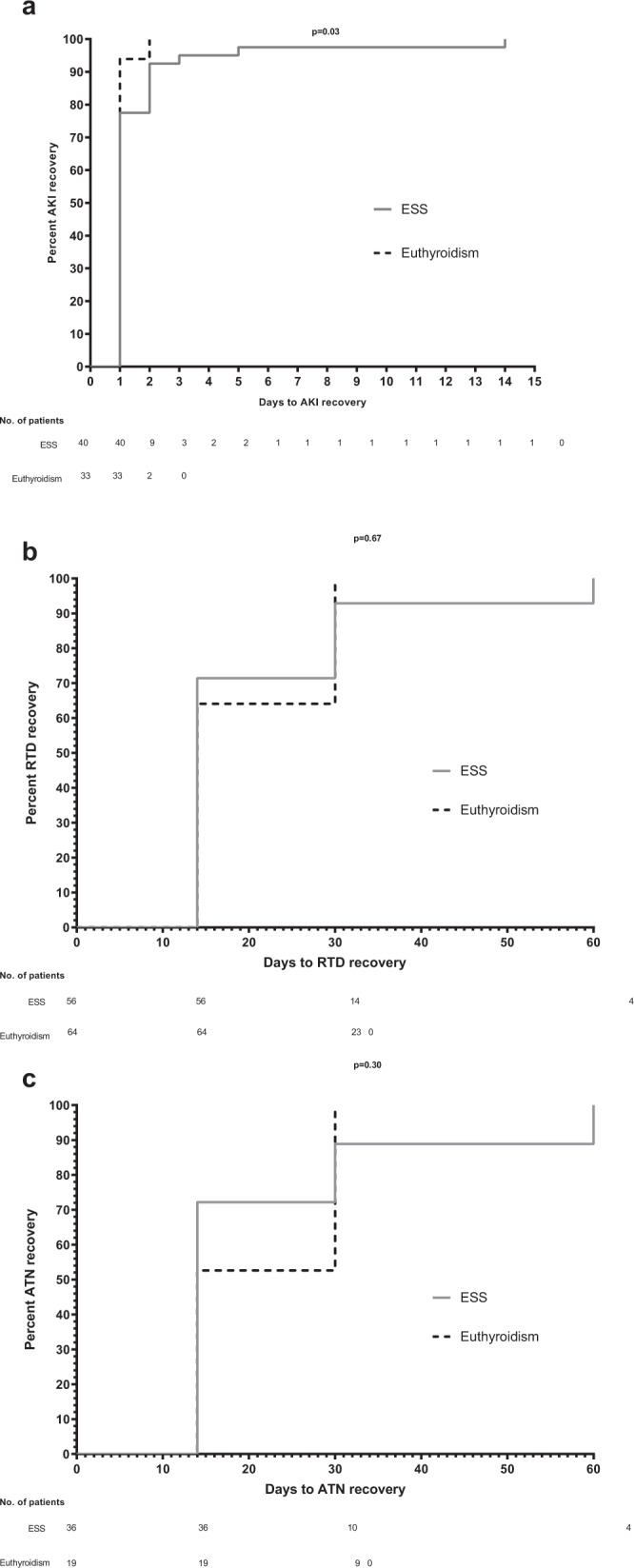
Kaplan-Meir analysis describing the recovery from AKI, RTD, and ATN. **a** Time needed to AKI resolution. AKI resolution according to ESS/euthyroidism: for the 40 patients with ESS and AKI the cumulative proportions of AKI resolution at the end of the following intervals were: 1 day, 77.5%; 2 days, 92.5%; 3 days, 95%, 5 days, 97.5%; 14 days, 100%. For the 33 patients with euthyroidism and AKI the cumulative proportions of AKI resolution at the end of the following intervals were: 1 day, 93.9%; 2 days, 100%. Log-rank test comparing the two Kaplan–Meier curves showed a *p* = 0.03. **b** Time needed to RTD resolution. RTD resolution according to ESS/euthyroidism: for the 56 patients with ESS and RTD the cumulative proportions of RTD resolution at the end of the following intervals were: 14 days, 71.4%; 30 days 92.8%; 60 days, 100%. For the 64 patients with euthyroidism and RTD the cumulative proportions of RTD resolution at the end of the following intervals were: 14 days, 64.1%; 30 days 100%. Log-rank test comparing the two Kaplan–Meier curves showed a *p* = 0.67. **c** Time needed to ATN resolution. ATN resolution according to ESS/euthyroidism: for the 36 patients with ESS and ATN the cumulative proportions of ATN resolution at the end of the following intervals were: 14 days, 72.2%; 30 days 88.9%; 60 days, 100%. For the 19 patients with euthyroidism and ATN the cumulative proportions of ATN resolution at the end of the following intervals were: 14 days, 52.6%; 30 days 100%. Log-rank test comparing the two Kaplan–Meier curves showed a *p* = 0.30.

The participants with euthyroidism showed an earlier recovery from AKI than those with ESS (*p* = 0.02) (Fig. 1a) while only a trend for an earlier recovery from RTD (Fig. 1b) and ATN was found (Fig. 1c).

## Discussion

Our longitudinal study describes in detail the relationship between clinical/metabolic factors at T1DM onset and thyroid hormones, with particular attention to the relationship between DKA-related kidney function impairment and ESS. Moreover, also the effect of ESS at T1DM onset on the recovery from kidney damage has been evaluated. Literature about this topic is scarce.

As expected, the participants with more severe T1DM onset presentation both in terms of metabolic derangement (DKA) and kidney function impairment (AKI, RTD and ATN) had an increased prevalence of ESS. It is known that AKI is associated with DKA severity.^5,6^ It is also known that ESS is associated with DKA severity.^10^ The remaining question was ESS is related to DKA severity or to AKI. Interestingly, we found that more than severe DKA, the ESS is associated with the maximal expression of kidney damage represented by the ATN which configures the shift of kidney damage from functional to intrinsic.^23^

Participants with ESS had lower BMI and higher weight loss. This outlines how undernutrition is one of the factors involved in the derangement of thyroid hormone also in DKA. In fact, it is known that fasting induces complex changes in thyroid hormones homeostasis including low serum T3 without increased TSH.^24^ This change has been suggested as a sort of beneficial adaptation during undernutrition in acute illness, decreasing the metabolic rate and reducing energy expenditure.^25^

In patients with DKA, higher levels of WBC and neutrophils compared with non-DKA T1DM patients have been described while no clear difference in lymphocytes count was evident.^26^

Interestingly, among our children with T1DM onset, participants with ESS had lower numbers of lymphocytes. This is in keeping with the recent observation by Grondman et al. of a correlation between abnormal thyroid function and lymphopenia in patients with severe infections, like bacterial sepsis and COVID-19.^27^ We can speculate that lymphopenia at T1DM onset could be a marker of the most severe forms of DKA which in turn could be strongly associated with ESS.

On the other hand, at follow-up, no differences in lymphocytes count were found comparing patients with and without ESS at T1DM onset. This strengthens the hypothesis that lymphopenia is a marker of T1DM onset severity with its disappearance when the acute phase is resolved.

In our work other determinants of FT3 levels were those related to the severity of DKA and to the severity of dehydration causing in turn RTD, AKI and in the most severe cases ATN such as triglycerides and creatinine levels among serum factors, and NGAL and UCa/Cr levels among urinary ones. Similarly, we found a direct association between FT4 and bicarbonates.

In DKA, the deficiency of insulin activates lipolysis in adipose tissue determining an increased release of free fatty acids, which accelerates the formation of very-low-density lipoproteins in the liver. In addition, reduced activity of lipoprotein lipase in peripheral tissue decreases their removal from the plasma, resulting in hypertriglyceridemia during episodes of DKA.^28^ Also cholesterol levels could be higher in DKA patients as a consequence of dehydration but not as often or as much as plasma triglycerides.^29^ Differently from the T1DM onset, at the follow-up visit we observed similar levels of triglycerides and a similar prevalence of abnormal triglycerides levels comparing participants with and without ESS (Table 1). On the other hand, unexpected findings were higher median cholesterol levels and a higher prevalence of hypercholesterolemia among participants with compared to those without ESS. We do not have a clear explanation, but it is possible that to normalize the cholesterol levels longer time is needed compared with the time of our follow-up visits.

The association between AKI and ESS has been described in adult patients by Iglesias et al. but not in children and not during DKA.^30^ In that adult population about 70% of those with AKI exhibited ESS.^30^ Interestingly, ESS recovered spontaneously as renal function improved.^30^ Moreover, ESS was not a prognostic factor in terms of hospital stay, recovery of renal function, need for renal replacement therapy by hemodialysis, development and degree of residual chronic renal failure and mortality in adults with AKI.^30^

Differently from this evidence,^30^ our pediatric findings suggest that ESS is also a prognostic factor in terms of time needed to recover from kidney damage. In fact, we showed that children with ESS had a slower recovery from AKI compared with those without ESS. This difference—in the short term—apparently has no clinical impact because all participants showed full recovery of renal function.

Patients having shown an AKI episode—especially in the severe forms—have per se an increased risk of chronic kidney disease.^8^ Moreover, it has been demonstrated that the timing of functional recovery from AKI is a factor associated with future adverse events^31^ and this evidence makes it mandatory that clinicians managing patients with AKI should consider the severity of the disease and the ensuing course and tailor their diagnostic and therapeutic interventions to facilitate rapid and complete recovery of kidney function.^32^

Considering this evidence and the fact that participants with AKI and ESS need more time to recover kidney function compared with participants with AKI without ESS, the possibility exists that in the long-term period, the patients with ESS and AKI could have an additional risk of chronic kidney disease compared with patients with AKI alone. Long-term follow-up studies are needed to clarify this point.

The fact that the ESS and AKI recovered in all the participants suggests that treatment of ESS could not be needed in these patients because the ESS could be simply expression of a greater severity of T1DM onset which in turn is associated with a greater risk of complications such as AKI. If a short course of treatment of ESS could accelerate the recovery from AKI so reducing the risk of chronic kidney disease remains to be established.

Anyway, treatment of ESS with T4 or T3 is still a matter of debate ad only a small number of randomized and controlled trials have been published and all report negative results in terms of clinical benefit.^33^

Besides the prognostic value, the pathophysiological mechanism of these findings will represent an interesting field of research.

In fact, previous studies suggested the ESS was mostly determined by the severity of acidosis in T1DM patients.^10,11^ Instead our data suggest that the overall severity of dehydration and undernutrition joined to the metabolic derangement contribute to a multifactorial action to the occurrence of ESS in T1DM onset pediatric patients.

The main limitation of our study is represented by the utilization of serum creatinine as an indicator of AKI in patients with DKA, despite acetoacetates, hyperglycemia, and glycosylated hemoglobin could falsely elevate measured creatinine.^34–36^ This effect is most pronounced with the Jaffe method (adopted in the present study) at low creatinine concentrations but is still observed with another enzymatic assay testing.^34,36^

For this reason, the AKI prevalence could be overestimated. The prevalence found in our study, however, is in line with a previous study adopting enzymatic^5^ serum creatinine measurement methods. Finally, serum creatinine is the only marker of renal function adopted in daily clinical practice, also in patients with DKA.

Another limitation is represented by the fact that DKA is associated with a systemic inflammatory condition that could lead to an increase in urinary NGAL levels regardless of the presence of AKI or renal tubular damage.^37^ In our study, however, we used also other urinary biomarkers to define the presence of AKI (i.e., urinary β2 microglobulin) and only two participants had isolated increase in urinary NGAL levels, so we think that the overestimation of the prevalence of renal tubular damage due to the presence of DKA-related inflammation is limited.

In conclusion, this is the first perspective observational study investigating the relationship between ESS and clinical and metabolic factors at T1DM onset. FT3 levels are inversely correlated with serum creatinine, serum triglycerides, urinary NGAL and UCa/Cr levels while FT4 levels are directly correlated with bicarbonates levels. The more severe the T1DM presentation, the higher the prevalence of ESS. In fact, patients with DKA, RTD, AKI, ATN have higher ESS prevalence compared with patients without these conditions. In multivariate logistic regression, the conditions most strongly associated with ESS are DKA and ATN. Patients with ESS show a lower recovery from AKI compared to those without AKI. ESS spontaneously disappears in all patients.

## Supplementary information

Supplementary figures

## Data Availability

The datasets generated during and/or analyzed during the current study are available from the corresponding author upon reasonable request.
