# Supplementary material for: Euthyroid sick syndrome and its association with complications of type 1 diabetes mellitus onset
Source: Pediatr Res. 2023 Jan 31;94(2):632–42. doi: 10.1038/s41390-023-02494-5 (PMC9887577; doi:10.1038/s41390-023-02494-5)

Supplemental figures legends

Supplemental Figure S1. ESS prevalence classifying the population on the basis of T1DM onset and kind of kidney involvement

Supplementary Figure S2A. Regression analysis describing the relationship between triglycerides and FT3.

Model r2=25.6 percent; p<0.001; correlation coefficient= -0.51. The regression is described by the equation y= 5,96175 - 0,408271*x. P value for intercept and slope was <0.001

Supplementary Figure S2B. Regression analysis describing the relationship between creatinine and FT3.

Model r2=18.5 percent; p<0.001; correlation coefficient= -0.42. The regression is described by the equation y= 0,142446 - 0,140689*x. P value for intercept was 0.2 and for slope was <0.001.

Supplementary Figure S2C. Regression analysis describing the relationship between NGAL and FT3.

Model r2=29.6 percent; p<0.001; correlation coefficient= -0.54. The regression is described by the equation y= 6,52485 - 1,08865*x. P value for intercept and slope was <0.001.

Supplementary Figure S2D. Regression analysis describing the relationship between UCa/Cr and FT3.

Model r2=31.7 percent; p<0.001; correlation coefficient= -0.56. The regression is described by the equation y= 1,12176 - 0,680746*x. P value for intercept and slope was <0.001.

Supplementary Figure S3. Regression analysis describing the relationship between bicarbonates and FT4.

Model r2=16.8 percent; p<0.001; correlation coefficient= 0.41. The regression is described by the equation y= 3,47412 + 1,13468*x. P value for intercept was 0.13 and for slope <0.001.

Supplemental Figure S1.

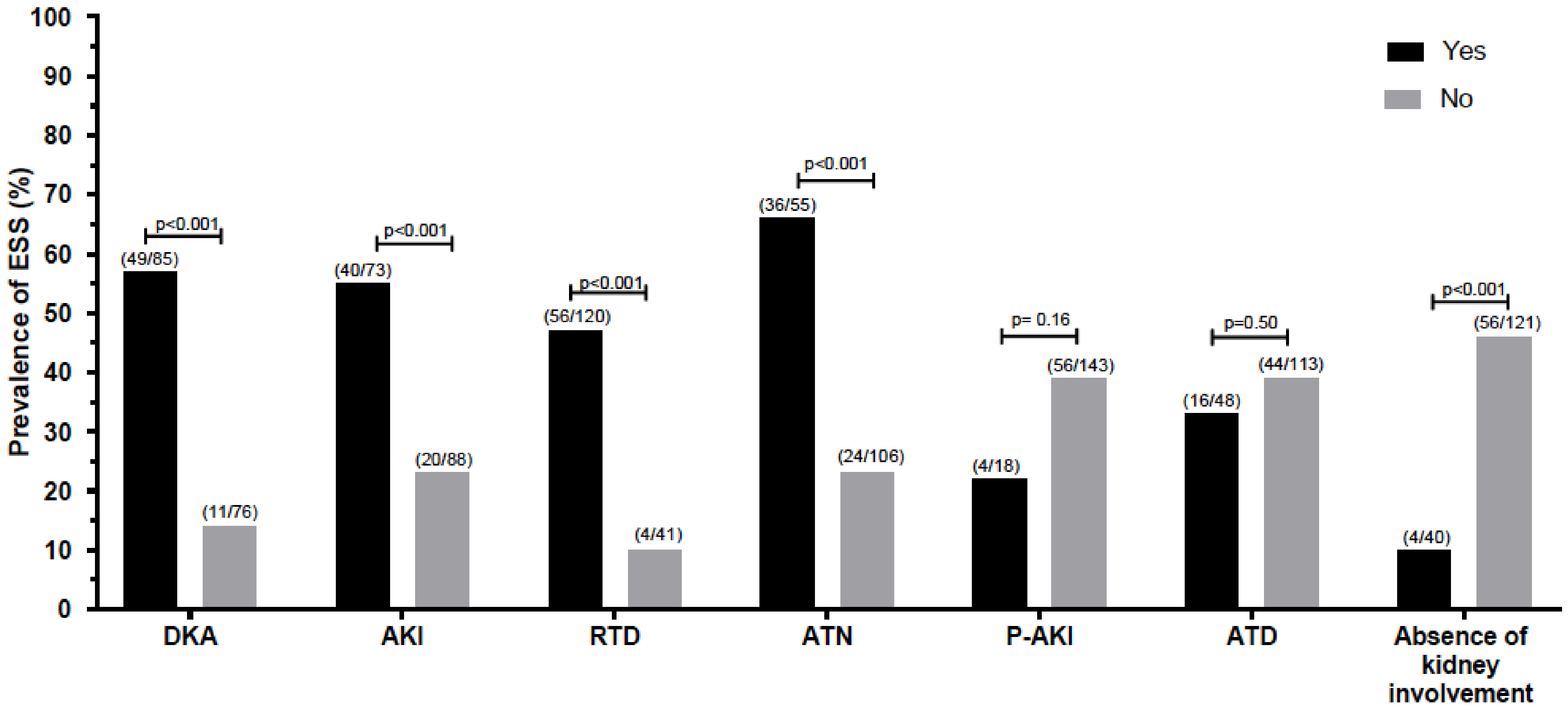

Supplemental Figure S2

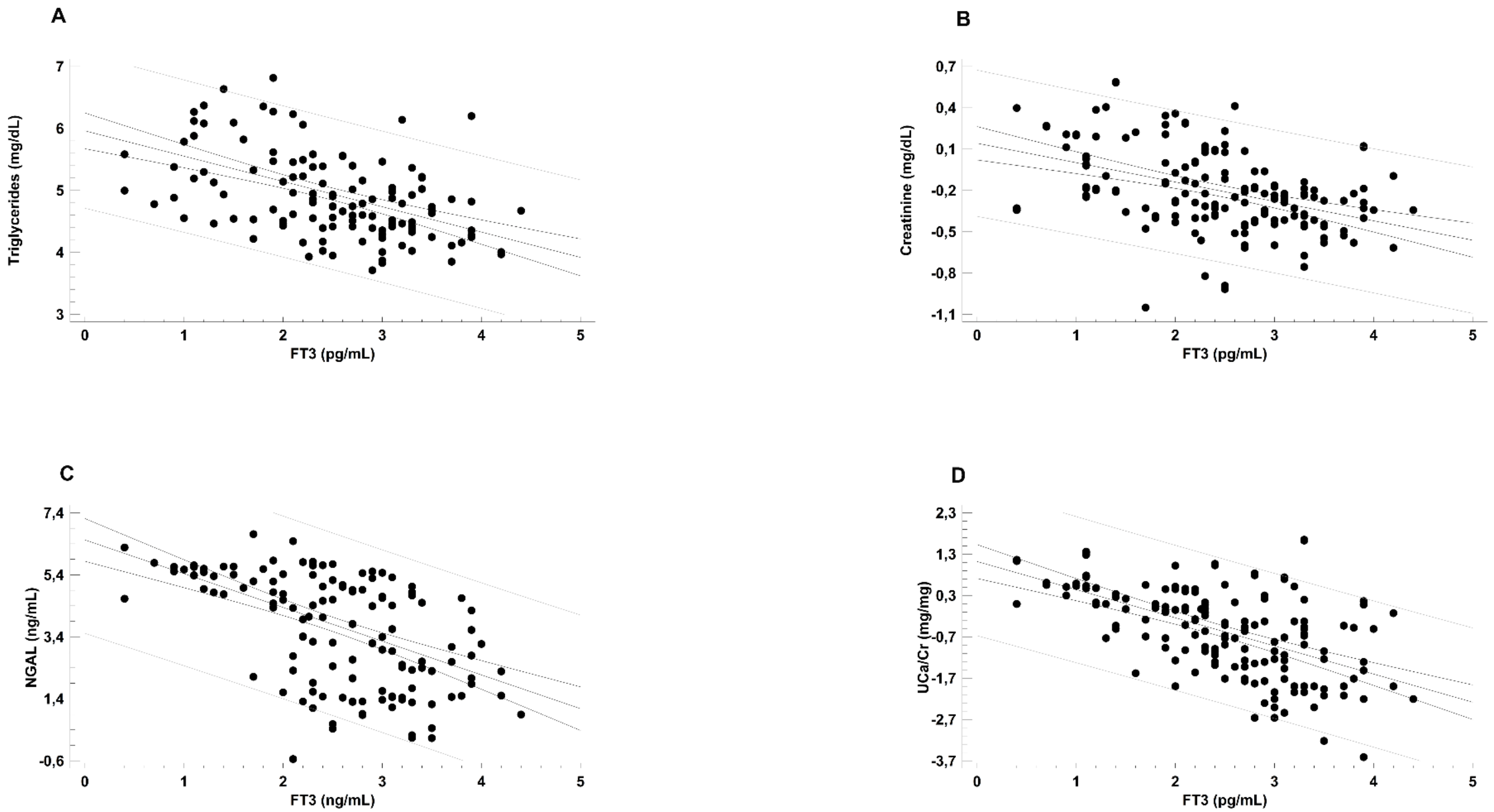

Supplemental Figure S3

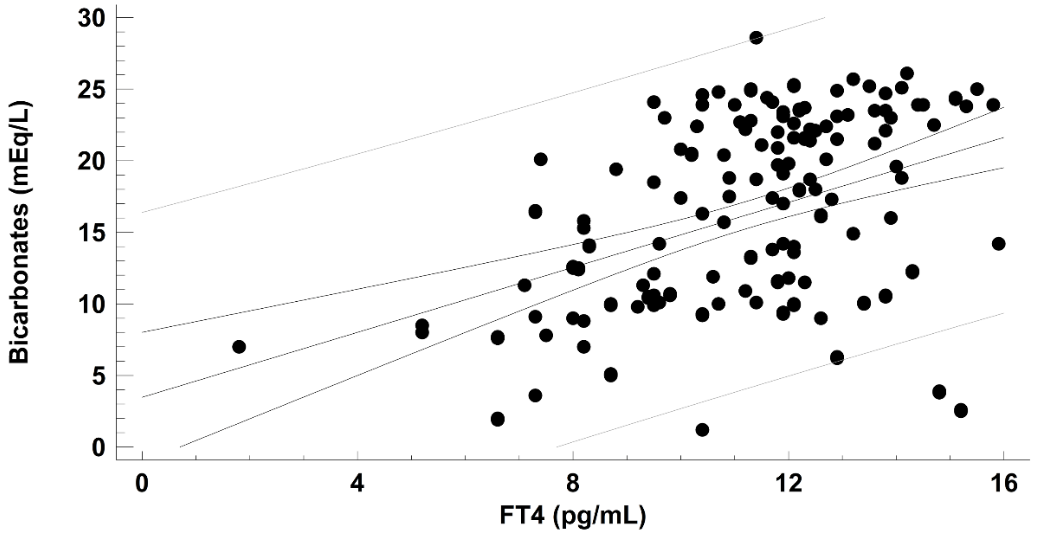

Supplement: Supplementary file 1 — Supplementary figures [file 41390_2023_2494_MOESM1_ESM.pdf]
